# Ultrasound-Assisted Enhancement of Germinated Sorghum: Multi-Omics Insights into Amino Acid and Carbohydrate Metabolic Regulation

**DOI:** 10.1016/j.ultsonch.2026.108013

**Published:** 2026-08-19

**Authors:** Qiao Huang, Yang Liu, Yiqiao Wang, Yiting Zhao, Jiarong Liu, Tao Xu, Yutang He, Guangchen Zhang, He Liu

**Affiliations:** a College of Food Science and Technology, Bohai University, Jinzhou, Liaoning 121013, China; b Department of Hepatobiliary Pancreatic Spleen Thyroid Surgery, General Hospital of Northern Theater Command, Shenyang, Liaoning 110000, China; c Rice Research Institute, Shenyang Agricultural University, Shenyang, Liaoning 110866, China; d Budweiser (Jinzhou) Beer Co., Ltd., Jinzhou 121100, Liaoning, China

**Keywords:** Sorghum, Ultrasound, Germination, Metabolomics, Transcriptomics

## Abstract

Germination represents a recognized strategy for improving the nutritional profile and promoting the enrichment of bioactive constituents in cereal crops. However, the regulatory mechanisms through which physical treatments such as ultrasound influence metabolic processes during germination remain insufficiently understood. This study investigates the effects of ultrasound-assisted germination (50 kHz, 240 W, 30 min) on amino acid and carbohydrate metabolism in sorghum while employing integrated metabolomic and transcriptomic approaches. Total protein and starch contents decreased during germination, with starch content in fully germinated ultrasound-treated grains (FG + U) declining to 344.27 mg/g. Protease and α-amylase activities increased during germination and were further improved by ultrasound treatment, suggesting increased mobilization of storage reserves during germination. Metabolomic analysis identified 110 amino acids and 49 carbohydrates as differentially accumulated metabolites under ultrasound treatment at the fully germinated stage, including six essential amino acids and increased D-glucose levels. Transcriptomic analysis revealed 7,791 genes shared between germination-related and ultrasound-responsive differentially expressed genes (DEGs). These genes were significantly enriched in metabolic pathways, including alanine, aspartate, and glutamate metabolism; glycolysis/gluconeogenesis; and the pentose phosphate pathway. Based on the Weighted gene co-expression network analysis (WGCNA)-derived gene modules, transcription factor–target gene network analysis further identified three highly connected transcription factors, including bHLH137 (*LOC8060932*), R-S (*LOC8056590*) and EIL1 (*LOC110434014*), as candidate hub transcription factors associated with amino acid and carbohydrate metabolism. These results provide systems-level insights into the transcriptional and metabolic responses associated with ultrasound-assisted germination and identify candidate regulatory components for future functional investigation.

## Graphical abstract

**Figure:**
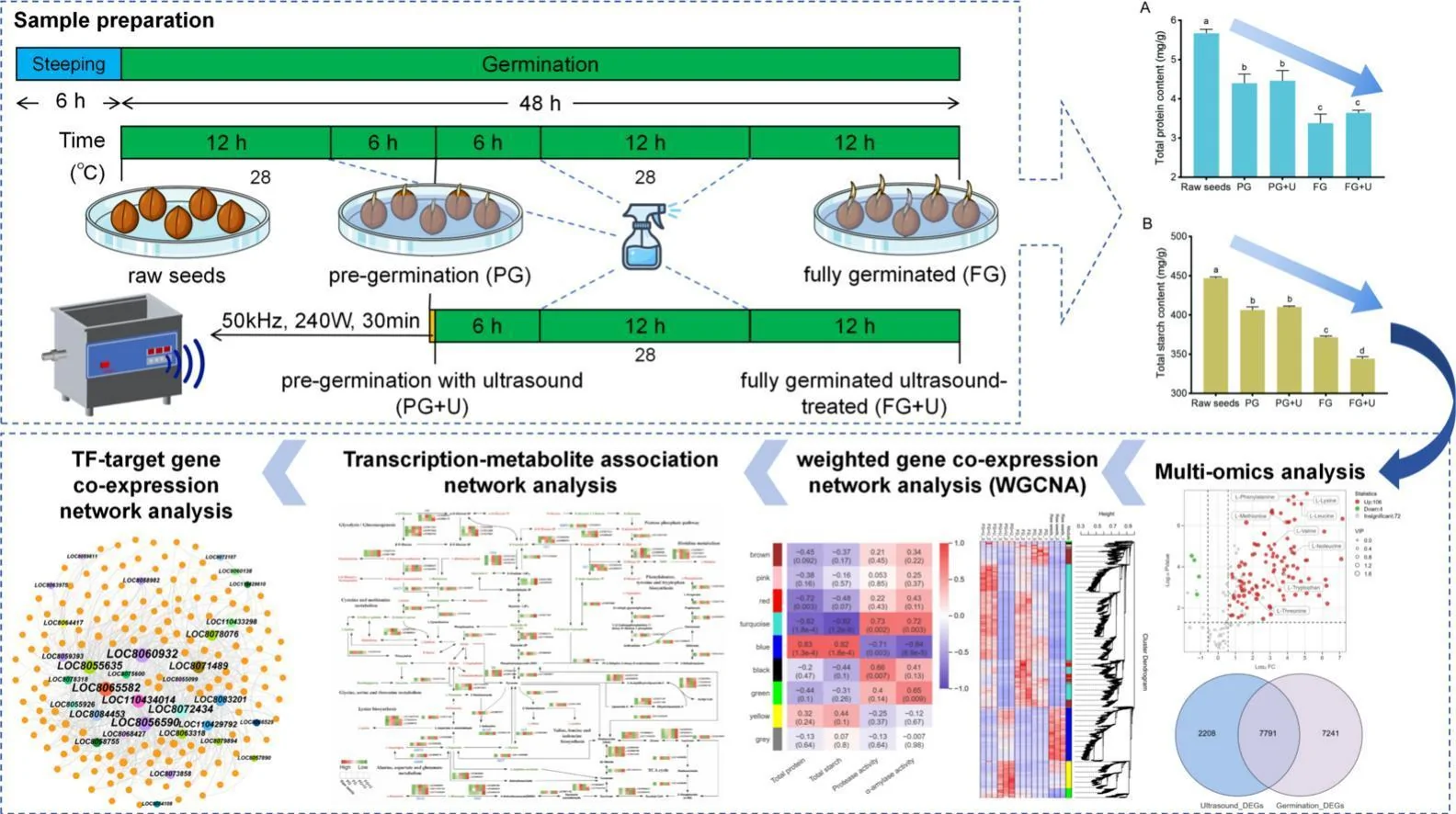

## 1 Introduction

Sorghum (*Sorghum bicolor* (L.) Moench), a major annual cereal crop, is widely recognized as the fifth most important cereal globally, after maize, rice, wheat, and barley [1]. It is traditionally used as animal feed; however, it has gained significant attention as a food grain due to its improved nutritional and functional properties [2]. Sorghum is consumed in various forms, including porridge, bread, and fermented beverages such as sorghum beer and liquor [3], [4], [5]. It is primarily composed of carbohydrates, mainly starch, and also contains dietary fiber, proteins, micronutrients, and diverse phytochemicals, particularly phenolic acids and flavonoids [6]. Given its nutritional and functional potential, appropriate processing methods are needed to further improve the quality and utilization of sorghum. Germination is considered an effective biological strategy for improving the nutritional and functional properties of cereal grains [7].

During germination, endogenous enzymes are activated to mobilize stored nutrients and support seedling growth. For example, increased amylolytic activity, particularly α-amylase activity, accelerates starch degradation and promotes the accumulation of reducing sugars in brown rice [8], [9]. Similarly, enhanced protein hydrolysis during the germination of lentil and fava bean increases the levels of free amino acids and peptides, contributing to improved protein digestibility and nutritional quality [10]. Germination can also increase the levels of bioactive compounds, including phenolics, dietary fiber, γ-aminobutyric acid (GABA), and minerals, while also decreasing antinutritional factors such as phytic acid [11]. Germination induces broad changes in carbohydrate, protein, and secondary metabolism. However, the extent of these responses depends on seed genotype, physiological status, germination duration, temperature, and moisture conditions, resulting in variable germination performance and providing a rationale for the use of appropriate pretreatment strategies [12].

Various physical treatments, including ultrasound, ultraviolet radiation, and non-thermal plasma, have been applied to improve germination performance. These technologies are considered eco-friendly and effective for seed processing applications [13]. Ultrasound, among these methods, represents a safe, cost-efficient, and environmentally friendly technique and has shown to improve germination and growth [14]. Ultrasound-assisted germination improves the nutritional and functional properties of plant-derived food materials by promoting protein structural transformation [15]. For example, ultrasonic treatment enhances soybean sprouting by improving water uptake, enzyme activity, and nutrient mobilization, and promotes germination performance in pumpkin seeds, resulting in higher germination rates [16], [17]. Recent transcriptomic studies indicate that ultrasound treatment is associated with coordinated transcriptional changes in stress-responsive signaling pathways and primary metabolic processes, particularly those involving amino acid and carbohydrate metabolism [18]. Beyond these transcriptional responses, ultrasound-enhanced germination is likely governed by multiple physiological and biochemical processes. For example, ultrasound-assisted germination promotes the accumulation of functional bioactive compounds, particularly polyphenols and flavonoids, enhancing the nutritional quality of germinated grains [19]. Despite these advantages, the effects of ultrasound are not universally positive. Its biological effects are highly dependent on treatment frequency, acoustic power, exposure duration, seed species, grain structure, and physiological status [20]. Appropriate ultrasound treatment may accelerate water absorption and metabolic activation, whereas excessive treatment can disrupt membrane integrity, induce oxidative stress, and even inhibit germination or subsequent seedling growth [21]. Despite these advances, previous studies have primarily focused on germination performance, enzyme activities, and nutritional changes, while the coordinated relationships between transcriptional responses and metabolic alterations during ultrasound-assisted germination remain poorly understood.

The relationships between transcriptional responses and metabolic changes associated with amino acid and carbohydrate metabolism during ultrasound-assisted germination remain largely unexplored. Recently, omics-based approaches have been increasingly used to investigate changes in seed metabolism during germination [22]. However, because amino acid and carbohydrate metabolism involves complex interactions between gene expression and metabolite accumulation, single-omics approaches may not fully elucidate these coordinated regulatory processes [23]. Therefore, integration of transcriptomic and metabolomic analyses provides a systems-level strategy for associating transcriptional regulation with metabolic alterations during ultrasound-assisted germination [23]. Weighted gene co-expression network analysis (WGCNA), a well-established systems biology framework, enables the identification of co-expressed gene modules associated with key metabolic traits and the discovery of potential regulatory genes governing complex biological processes. This makes it particularly suitable for deciphering the molecular networks underlying ultrasound-responsive metabolic regulation [24], [25].

Therefore, this study aims to investigate the transcriptional and metabolic changes associated with ultrasound-assisted germination, with particular emphasis on amino acid and carbohydrate metabolism in sorghum. We hypothesized that ultrasound modulates amino acid and carbohydrate metabolism through coordinated changes in gene expression and metabolite accumulation during germination. To test this hypothesis, integrated transcriptomic and metabolomic analyses, together with WGCNA and transcription factor analysis, were employed to identify the key regulatory genes and molecular networks underlying ultrasound-responsive metabolic regulation. This study provides new mechanistic insights into ultrasound-assisted germination and supports the development of nutrient-enriched sorghum-based functional foods.

## 2 Materials and methods

### 2.1 Plant material preparation

Six sorghum varieties (Liaonian 3, Liaonian 10, Liaonian 16, Liaoza 19, Liaoza 52, and Liaoza 61) were obtained from the experimental base of the Liaoning Academy of Agricultural Sciences, Shenyang, Liaoning Province, China. After dehulling, the grains were stored at 4 °C until further processing.

Sorghum grains with uniform size were selected, followed by their sterilization using a freshly prepared 0.1% aqueous sodium hypochlorite solution for 30 min. Subsequently, sorghum grains (200 g) were rinsed with deionized water and soaked in distilled water with the grains completely submerged for 6 h. After removal of excess water, the grains were evenly spread in germination trays and incubated in the dark at 28 ℃ under 90% relative humidity. During germination, the grains were systematically sprayed with freshly prepared deionized water at intervals of 12 h to sustain an appropriate level of surface moisture. These six varieties were initially screened by comparing the germination lengths between fully germinated (FG) and fully germinated ultrasound-treated (FG + U) grains. The samples of Liaonian 3 were collected at predetermined intervals of 0 h, 18 h, and 48 h during germination and designated as raw seeds, pre-germination (PG), and fully germinated (FG), respectively. Surface moisture was removed using filter paper before freeze-drying. The samples were then transferred and stored at − 80 °C for long-term preservation. For ultrasonic treatment, grains that had been germinated for 18 h were removed from the incubator and transferred into beakers containing deionized water. Ultrasonication was performed in a 28 °C ultrasonic water bath at 50 kHz and 240 W for 30 min under continuous operation. The ultrasonic parameters were selected based on a previous study [26]. Following treatment, one portion of the samples was directly freeze-dried and designated as PG + U. The other samples were returned to the incubator for continued germination until 48 h, followed by freeze-drying and designated as FG + U.

### 2.2 Determination of protein and starch contents

Total protein content of sorghum grains was determined using the Kjeldahl method with minor modifications according to Batool et al. [27]. A 2 g sorghum sample was subjected to acid digestion with 0.4 g CuSO_4_, 6 g K_2_SO_4_, and 20 mL H_2_SO_4_ at 420 °C for 2 h. Nitrogen content was then determined with an automated Kjeldahl-based nitrogen analyzer while using 0.1 mol/L HCl as the titration standard. A conventional factor of 6.25 (nitrogen-to-protein conversion factor) was applied to estimate protein content.

Total starch content of sorghum grains was quantified with a commercially available starch assay kit (AKSU015M, 120 T/100S). Powdered samples were dispersed in 5 mL of deionized water and maintained in a boiling water bath under controlled heating conditions for 60 min. The suspensions were subsequently centrifuged at 4000 × g for 20 min to facilitate phase separation. Supernatant (2 mL) was thoroughly mixed with 3 mL of freshly prepared anthrone reagent (0.4% in concentrated H_2_SO_4_) and maintained at 90 °C in a water bath for 20 min. The absorbance of the treated mixture was measured at 620 nm. All measurements were performed using three biological replicates.

### 2.3 Enzyme activity assays

The activities of protease and α-amylase were determined using assay kits (BC2290 and BC0610, Beijing Solarbio Science & Technology Co., Ltd) according to the manufacturer’s instructions.

For protease activity, 1.0 g of sample was homogenized with extraction buffer at a ratio of 1:5 (w/v) under ice-cold conditions and centrifuged at 10,000 rpm for 10 min at 4 °C. The supernatant was incubated with the substrate at 40 °C for 10 min, followed by color development at 40 °C for 20 min. Absorbance was measured at 680 nm, and protease activity was calculated using a standard curve of tyrosine.

For α-amylase activity, 0.2 g of sample was homogenized with 1.5 mL of distilled water, extracted at room temperature for 15 min, and centrifuged at 6,000 × g for 10 min. The supernatant was diluted to 10 mL and incubated with the substrate at 70 °C for 15 min. Following reacting with 3,5-dinitrosalicylic acid in a boiling water bath for 10 min, absorbance was measured at 540 nm. α-amylase activity was calculated using a glucose standard curve. Both enzyme activities were expressed as U/g sample.

### 2.4 Metabolomic profiling and data analysis

Metabolomic profiling was performed on FG and FG + U sorghum samples. FG and FG + U samples represented fully germinated sorghum grains collected at the same germination stage, with the principal difference between the two groups being the application of ultrasound treatment. Three independent biological replicates were used for each experimental group to ensure the analytical data reproducibility and reliability. Each sample aliquot (100 mg) was ground to a fine powder under liquid nitrogen, extracted with 0.5 mL of ice-cold 80% (v/v) methanol, and subjected to vigorous mixing. The extracts were then incubated on ice for 5 min, followed by centrifugation at 15,000 × g for 20 min at 4 °C to pellet the insoluble material and recover the supernatant. The supernatants were analyzed while employing an LC–MS/MS platform. Data were acquired on an ExionLC™ AD liquid chromatography system coupled to a QTRAP® 6500 + mass spectrometer (SCIEX). A quality control (QC) sample was prepared by mixing aliquots from each biological sample and used for quality assessment throughout analysis. These QC aliquots were periodically injected throughout the LC–MS/MS run to evaluate both the stability of the instrumentation and the reproducibility of the analytical procedure. An XSelect HSS T3 column (2.1 × 150 mm, 2.5 μm) was employed for chromatographic profiling. Separation of analytes was performed using a 20-min gradient program delivered at 0.4 mL/min. Mass spectrometric detection was carried out under both positive and negative ionization conditions.

A binary solvent system was employed; the mobile phase system comprised aqueous 0.1% formic acid (FA-A) and acetonitrile with 0.1% formic acid (FA-B). The gradient profile began at 2% FA-B and was held constant for the first 2 min. The proportion of FA-B was increased linearly to 100% by 17 min, held momentarily, and then restored to the initial composition at 17.1 min. Re-equilibration was performed at 2% FA-B for the remainder of the analytical run until 20 min. Dynamic multiple reaction monitoring (dMRM) acquisition was performed under the following conditions: for positive ionization, the instrument was operated with an ion spray voltage of 5500 V, curtain gas at 35 psi, collision gas set to medium, source temperature of 550 °C, and ion source gases 1 and 2 at 60 psi. For negative ion mode, only the ion spray voltage was adjusted to − 4500 V, while all other parameters were unchanged. A widely targeted metabolomics approach based on dMRM was employed for metabolite profiling.

Metabolite identification was performed using the NovoDB (Novogene Database), a proprietary metabolite database established with authentic reference standards on the SCIEX QTRAP® 6500 + mass spectrometry platform. Metabolites were identified by matching precursor ion (Q1), product ion (Q3), retention time (RT), declustering potential (DP), and collision energy (CE). Metabolite identification confidence was classified as MSI Level 1 because all metabolites were identified using authentic reference standards. The Kyoto Encyclopedia of Genes and Genomes (KEGG; http://www.kegg.jp/kegg/compound/), the LipidMaps database (http://www.lipidmaps.org/), and the Human Metabolome Database (HMDB; http://www.hmdb.ca/) were subsequently used for metabolite annotation, functional classification, and pathway enrichment analysis. Peak intensity data were processed using MetaboAnalystR in R. Metabolites with fold change (FC) ≥ 1.5 or ≤ 0.67, *p* < 0.05, and variable importance in projection (VIP) > 1.0 were considered differentially accumulated metabolites (DAMs). The OPLS-DA model was validated using 200 permutation tests to assess model overfitting. Metabolic pathway interpretation and enrichment analysis were performed using the KEGG pathway database (http://www.kegg.jp/kegg/pathway.html). Metabolite set enrichment analysis (MSEA) was conducted using hypergeometric testing to determine statistical significance.

### 2.5 RNA sequencing and transcriptomic analysis

RNA quality was evaluated with an RNA Nano 6000 Assay Kit on an Agilent Bioanalyzer 2100 platform (Agilent Technologies, CA, USA), and intact total RNA served as the starting material for library preparation. In brief, polyadenylated mRNA was selectively captured via poly-T oligo-conjugated magnetic beads, thermally fragmented in First Strand Synthesis Reaction Buffer (5X), and converted into first-strand cDNA by means of random hexamer primers and M−MuLV reverse transcriptase (RNase H − ). Second-strand cDNA synthesis was subsequently completed using DNA Polymerase I and RNase H, followed by end repair, 3′ adenylation, and ligation of hairpin loop adaptors. cDNA fragments of 370–420 bp were selected using the AMPure XP system (Beckman Coulter, Beverly, USA). Libraries were amplified with Phusion High-Fidelity DNA Polymerase, universal PCR primers, and index primers, purified with AMPure XP beads, and subsequently evaluated using the Agilent Bioanalyzer 2100 system.

Gene expression quantification was performed using FeatureCounts v1.5.0-p3, and FPKM values were subsequently computed taking both gene length and read counts into account. To minimize false discoveries, raw *p*-values derived from DESeq2-based differential expression analysis were corrected using the Benjamini–Hochberg procedure for FDR control. Differentially expressed genes (DEGs) were defined as genes with an FDR < 0.05 and |log2FoldChange| ≥ 1. Gene Ontology (GO) and KEGG pathway enrichment analyses were performed using the OmicShare platform (https://www.omicshare.com/). WGCNA was conducted using the Metware Cloud platform (https://cloud.metware.cn), with a minimum module size of 50 genes and a module merge dissimilarity threshold of 0.25. Correlation analysis between the MEturquoise module eigengene and physiological traits was performed using Pearson and Spearman correlation analyses. Bootstrap resampling (2000 iterations) with bias-corrected and accelerated (BCa) 95% confidence intervals was applied to evaluate the robustness of Pearson correlation coefficients. Stratified bootstrap resampling was performed according to experimental groups. A leave-one-out sensitivity analysis was conducted to assess the stability of correlations between the MEturquoise module eigengene and physiological traits. Each sample was sequentially removed, and Pearson correlation coefficients were recalculated using the remaining samples.

### 2.6 RT-qPCR validation

Selected DEGs were validated at the transcript level by RT-qPCR, with *ACT*-1 serving as the internal reference gene. Primer sequences used for amplification are presented in Table S1, and relative expression levels were derived using the 2^-ΔΔCt calculation method. Each biological replicate was evaluated in triplicate through independent technical replicates to confirm analytical accuracy and reproducibility.

### 2.7 Wgcna-based TF-target gene co-expression network analysis

The turquoise co-expression module identified through WGCNA, containing 3,744 genes, was used for network construction. Transcription factors (TFs) were screened based on module membership (MM) ≥ 0.85, gene significance (GS) ≤  − 0.88, and *p* < 0.01, resulting in the identification of 33 candidate TFs. Target genes were selected using MM ≥ 0.85, GS ≤  − 0.85, and *p* < 0.01. These stringent thresholds were used to prioritize genes with strong module membership and trait association. Genes associated with amino acid metabolism, carbohydrate metabolism, and energy metabolism were identified based on KEGG BRITE classification (B class), yielding 159 candidate target genes. Pearson correlation analysis was performed using the corr.test() function in R v3.5.1. Gene pairs with correlation coefficients ≥ 0.95 and *p* < 0.01 were considered significantly co-expressed. This stringent correlation threshold was used to retain high-confidence co-expression relationships. TFs or target genes that did not form significant co-expression relationships were excluded from the final network. After filtering, the final co-expression network consisted of 32 TFs, 150 target genes, and 841 interaction edges. Visualization of the network was carried out using Gephi v0.10.1.

### 2.8 Statistical analysis

Data are presented as mean ± standard deviation (SD) from three biological replicates. Statistical significance was determined using one-way analysis of variance (ANOVA) followed by Tukey’s post hoc test for multiple comparisons. Statistical analyses were performed using SPSS v27.0 (IBM, Armonk, NY, USA), with *p* < 0.05 considered statistically significant.

## 3 Results

### 3.1 Changes in total protein and starch contents during germination and ultrasonic treatment

To evaluate the effect of ultrasound on sorghum germination and select a suitable variety for subsequent experiments, the germination lengths of six sorghum varieties under FG and FG + U treatments were compared (Fig. S1). The results showed that ultrasound promoted sprout elongation in all varieties, with Liaonian 3 displaying the most pronounced response and therefore being selected for subsequent analyses.

**Fig. 1:**
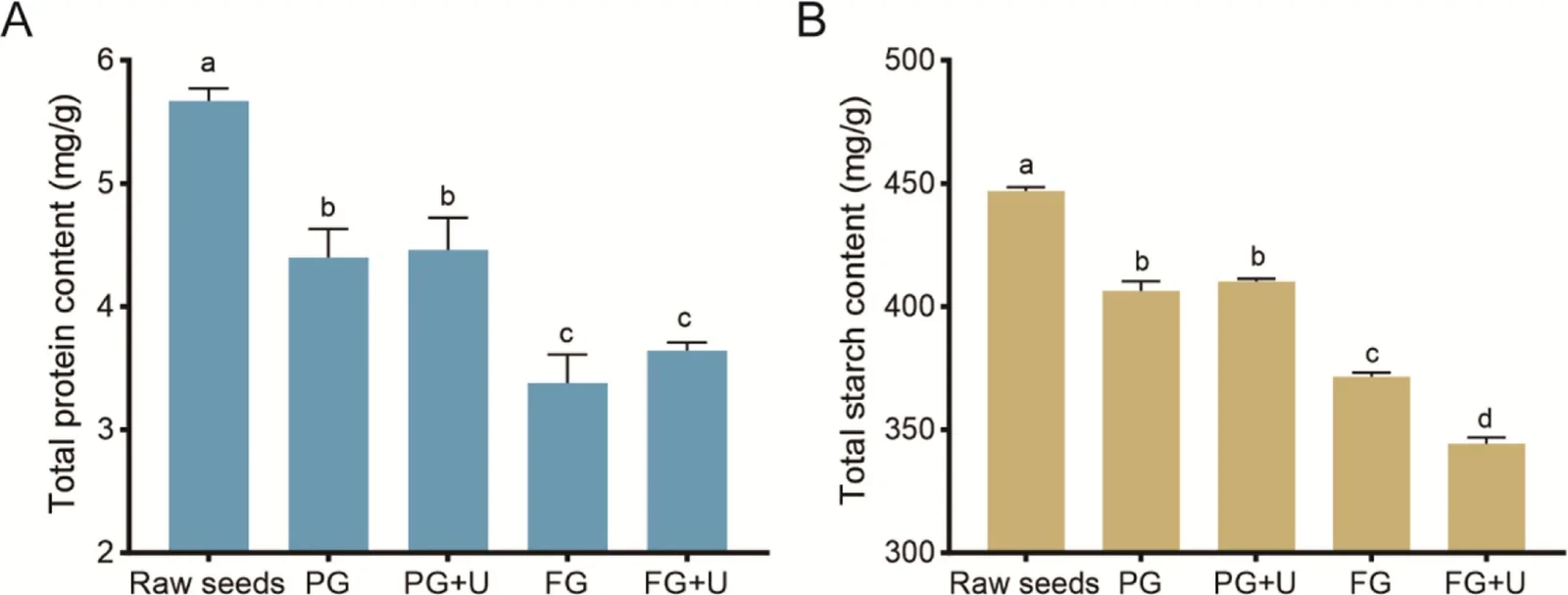
Quantification of (A) total protein content and (B) total starch content across raw seeds, PG, PG + U, FG, and FG + U sample groups. Data are presented as mean ± SD (n = 3). Different letters indicate significant differences at *p* < 0.05.

Raw seeds exhibited the highest total protein content (Fig. 1A), indicating that storage proteins are abundant in non-germinated grains. Protein content decreased significantly after pre-germination (PG) and pre-germination with ultrasound (PG + U), reaching comparable levels. This suggests that protein degradation is initiated during early germination. A further reduction was observed in fully germinated (FG) and fully germinated ultrasound-treated (FG + U) samples, with the lowest protein levels recorded at this stage. This pattern is consistent with extensive mobilization of storage proteins during germination to support embryo development. Protease activity increased during germination and was higher in ultrasound-treated samples than in the corresponding untreated groups (Fig. S2). Similarly, total starch content followed a declining trend across treatments (Fig. 1B). Raw seeds contained the highest starch content, while PG and PG + U treatments resulted in an intermediate reduction, reflecting early activation of starch-degrading enzymes. As germination progressed, the starch content in FG samples declined significantly. Compared with FG, the FG + U group exhibited a significantly lower starch content (344.27 mg/g, *p* < 0.05). Correspondingly, α-amylase activity was higher in ultrasound-treated samples than in the respective untreated groups throughout germination (Fig. S2). These results suggest that ultrasound treatment is associated with changes in protein and starch metabolism during germination, which may influence amino acid and carbohydrate metabolic profiles in sorghum.

### 3.2 Metabolomic alterations in FG and FG + U sorghum grains

**Fig. 2:**
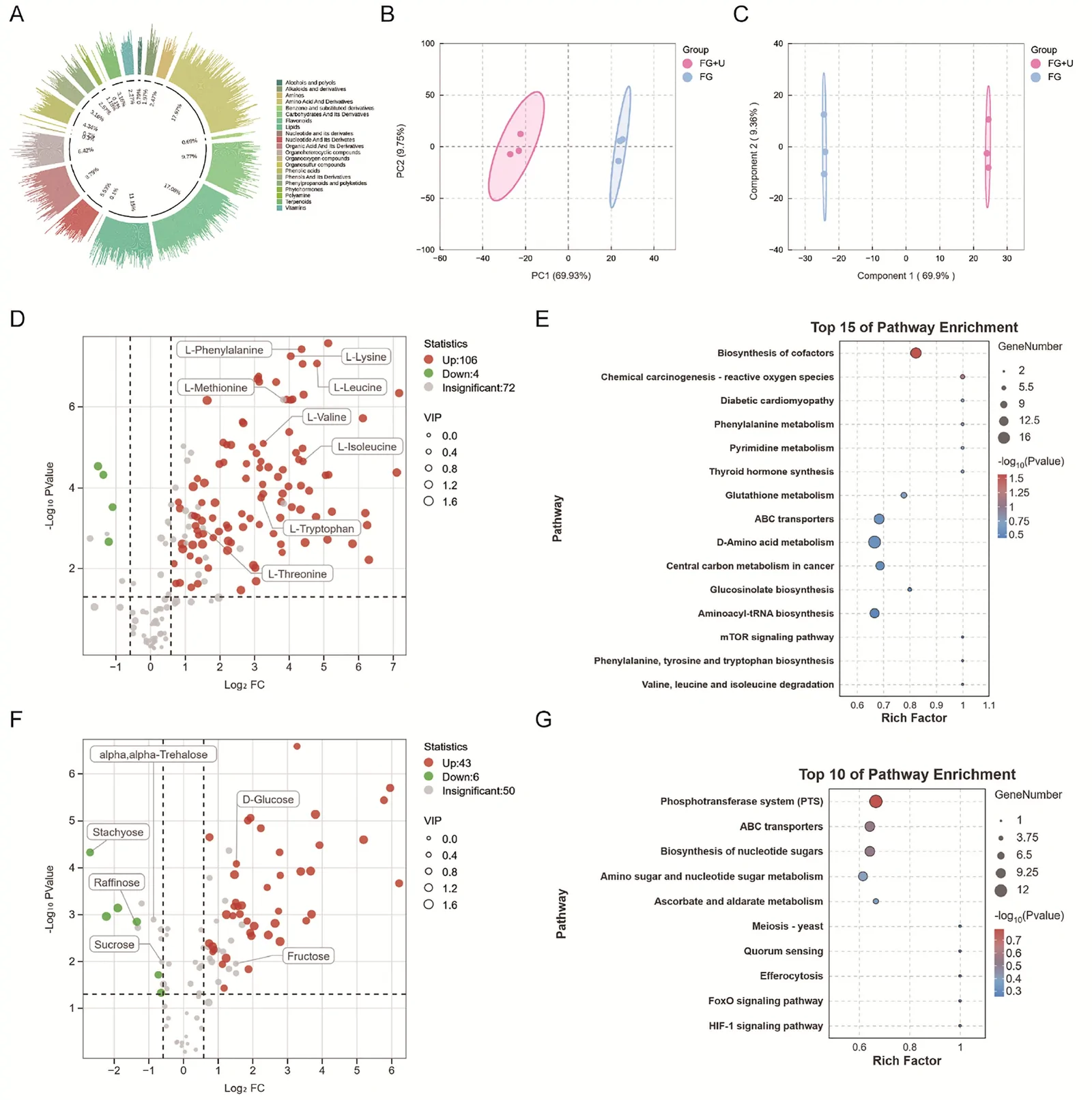
Preliminary metabolomic analysis of FG + U versus FG sorghum samples. (A) Classification. (B) PCA score plot. (C) OPLS-DA score plot of identified metabolites. Volcano plot of differentially accumulated (D) amino acid and (F) carbohydrate metabolites. KEGG pathway enrichment analysis of (E) amino acid-related and (G) carbohydrate-associated metabolites.

To investigate metabolic alterations associated with ultrasound-assisted germination during germination, metabolomic profiling was performed on FG and FG + U sorghum samples using LC-MS/MS. As shown in Fig. 2A, metabolite annotation revealed broad classification across multiple chemical groups, including amino acids and derivatives (17.97%), flavonoids (17.08%), lipids (11.15%), carbohydrates and its derivatives (9.77%), and organic acid and its derivatives (8.79%). PCA showed clear separation of the metabolic profiles between FG and FG + U along the first two principal components (PC1 and PC2), which accounted for 69.93% and 9.75% of the total metabolic variance, respectively (Fig. 2B). Consistent with the PCA results, OPLS-DA showed clear separation between FG and FG + U samples, while intra-group clustering remained tight (Fig. 2C), indicating differences in the overall metabolic profiles between the two groups. The robustness of the OPLS-DA model was further evaluated by permutation testing. As shown in Fig. S3, the original model showed higher explanatory and predictive values (R^2^Y = 1 and Q^2^ = 0.987) than the randomly permuted models, indicating good discriminatory performance for the current dataset.

To further explore metabolic changes, a clustered heat map (Fig. S4) was used to compare amino acid and carbohydrate profiles between the two groups. Most carbohydrate metabolites showed higher relative abundance in FG + U than in FG, indicating differences in carbohydrate-associated metabolite accumulation patterns between the two groups. Similarly, amino acid-related metabolites generally exhibited higher relative abundance in FG + U, suggesting differences in amino acid-associated metabolite accumulation profiles between the two groups. Volcano plot analysis identified 182 amino acid-related metabolites (Fig. 2D), among which 106 were significantly up-regulated, and 4 were significantly down-regulated in FG + U relative to FG. Several annotated amino acid metabolites, including L-phenylalanine, L-lysine, L-tryptophan, L-valine, L-leucine, and L-isoleucine, showed increased abundance in FG + U samples, indicating differential accumulation of amino acid-related metabolites between the two groups. KEGG pathway enrichment analysis showed that the differential metabolites were primarily associated with amino acid-related pathways. Significant enrichment was observed in phenylalanine metabolism, D-Amino acid metabolism, aminoacyl-tRNA biosynthesis, and phenylalanine, tyrosine, and tryptophan biosynthesis (Fig. 2E), suggesting that these pathways were associated with the metabolic differences observed between FG + U and FG samples. Similarly, volcano plot analysis of 99 carbohydrate-related metabolites identified 43 up-regulated and 6 down-regulated metabolites (Fig. 2F). D-glucose was significantly increased, whereas stachyose was significantly decreased. KEGG pathway enrichment analysis (Fig. 2G) identified significant enrichment of differential metabolites associated with the phosphotransferase system (PTS), nucleotide sugar biosynthesis, and ABC transporters.

### 3.3 Transcriptomic profiling of germinated sorghum under ultrasound treatment

To characterize transcriptomic changes induced by ultrasound treatment during sorghum germination, five sample groups: raw seeds, PG, FG, PG + U, and FG + U, were subjected to RNA-seq analysis. A total of 98.33 G clean reads were generated. The number of clean reads per sample ranged from 42,733,840 to 51,237,726, with a total of 655,452,808 reads across all samples. The average GC content was 54.09%, and Q30 bases accounted for 92.17% of total reads (Table S2).

**Fig. 3:**
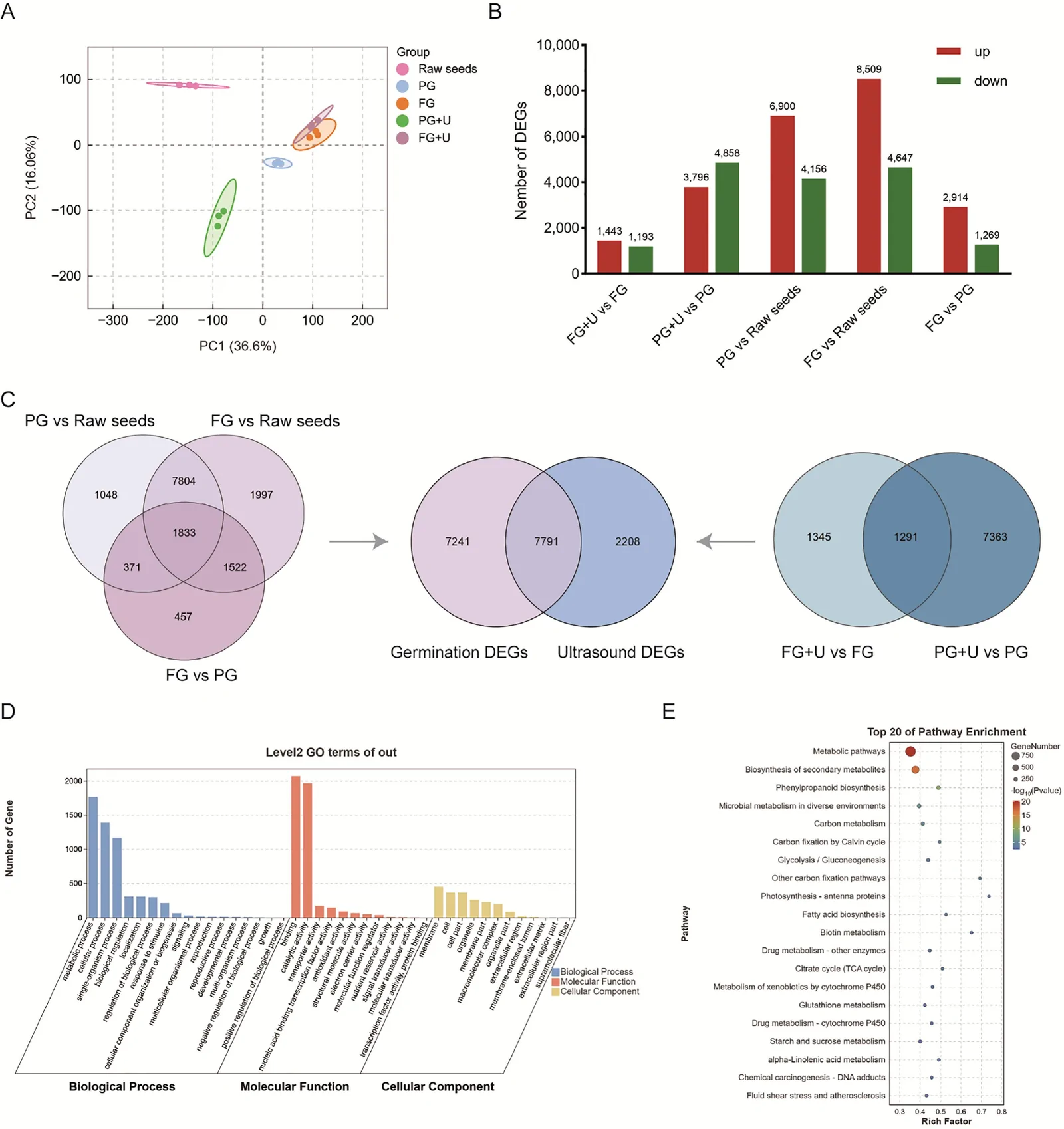
Preliminary transcriptomic analysis of raw seeds, PG, FG, PG + U, and FG + U groups. (A) PCA score plots. (B) DEGs identified across treatments. (C) Venn diagram of DEGs among different pairwise comparisons. (D) GO functional enrichment analysis of 7,791 DEGs. (E) KEGG pathway enrichment analysis of 7,791 DEGs.

PCA revealed clear transcriptional separation among raw seeds, pre-germinated (PG), fully germinated (FG), and their respective ultrasound-treated counterparts (PG + U and FG + U) (Fig. 3A). PC1 (36.8%) and PC2 (16.8%) together explained more than half of the total variance, indicating substantial transcriptional reprogramming during germination. Ultrasound treatment further shifted transcriptomic profiles away from their corresponding non-treated groups, particularly in PG + U and FG + U, suggesting an additional regulatory effect of ultrasound during germination.

### 3.4 Identification and functional characterization of DEGs

To identify genes associated with germination and ultrasound treatment, DEGs were analyzed across five comparison groups (Fig. 3B). A large number of DEGs were identified across all comparisons, indicating transcriptional remodeling during germination and under ultrasonic treatment. In PG vs raw seeds, 11,056 DEGs were detected, including 6,900 up-regulated and 4,156 down-regulated genes. In FG vs raw seeds, 13,156 DEGs were identified (8,509 up-regulated and 4,647 down-regulated), indicating stronger transcriptional changes during full germination. Comparison of FG vs PG revealed 4,183 DEGs (2,914 up-regulated and 1,269 down-regulated), reflecting developmental progression-dependent transcriptional shifts. Ultrasound treatment induced further transcriptional changes. In PG + U vs PG, 8,654 DEGs were identified (3,796 up-regulated and 4,858 down-regulated). FG + U vs FG yielded 2,636 DEGs (1,443 up-regulated and 1,193 down-regulated), indicating a measurable regulatory effect of ultrasound during germination. To validate RNA-seq results, five representative genes were randomly chosen for RT-qPCR-based validation. The expression trends revealed by RT-qPCR were in close concordance with the RNA-seq data (Fig. S5), substantiating the accuracy and reliability of the transcriptomic dataset.

Venn diagram analysis identified both shared and unique DEGs among comparisons (Fig. 3C). A total of 7,791 genes were shared between germination-related and ultrasound-related DEGs, indicating a substantial overlap in transcriptional responses. These shared DEGs were selected for further functional analysis to explore common regulatory mechanisms underlying germination and ultrasonic treatment. GO functional enrichment analysis of the 7,791 shared DEGs was performed at level 2 (Fig. 3D). The enriched GO terms were predominantly assigned to metabolic process, cellular process, and single-organism process within the Biological Process category. Binding and catalytic activity constituted the major Molecular Function terms, indicating extensive biochemical activity. Genes were largely distributed among membrane, cell, and cell part terms in the Cellular Component category. These results indicate that the shared gene set is strongly associated with metabolic activity and cellular function during germination and ultrasound exposure. KEGG pathway enrichment analysis of the shared DEGs (Fig. 3E) revealed significant enrichment in metabolic pathways and biosynthesis of secondary metabolites. Pathways related to carbon metabolism, including glycolysis/gluconeogenesis and carbon fixation, as well as amino acid and lipid metabolism, were significantly represented, indicating coordinated regulation of central metabolic networks during germination and ultrasonic treatment.

### 3.5 Wgcna-based co-expression network and module–trait analysis

**Fig. 4:**
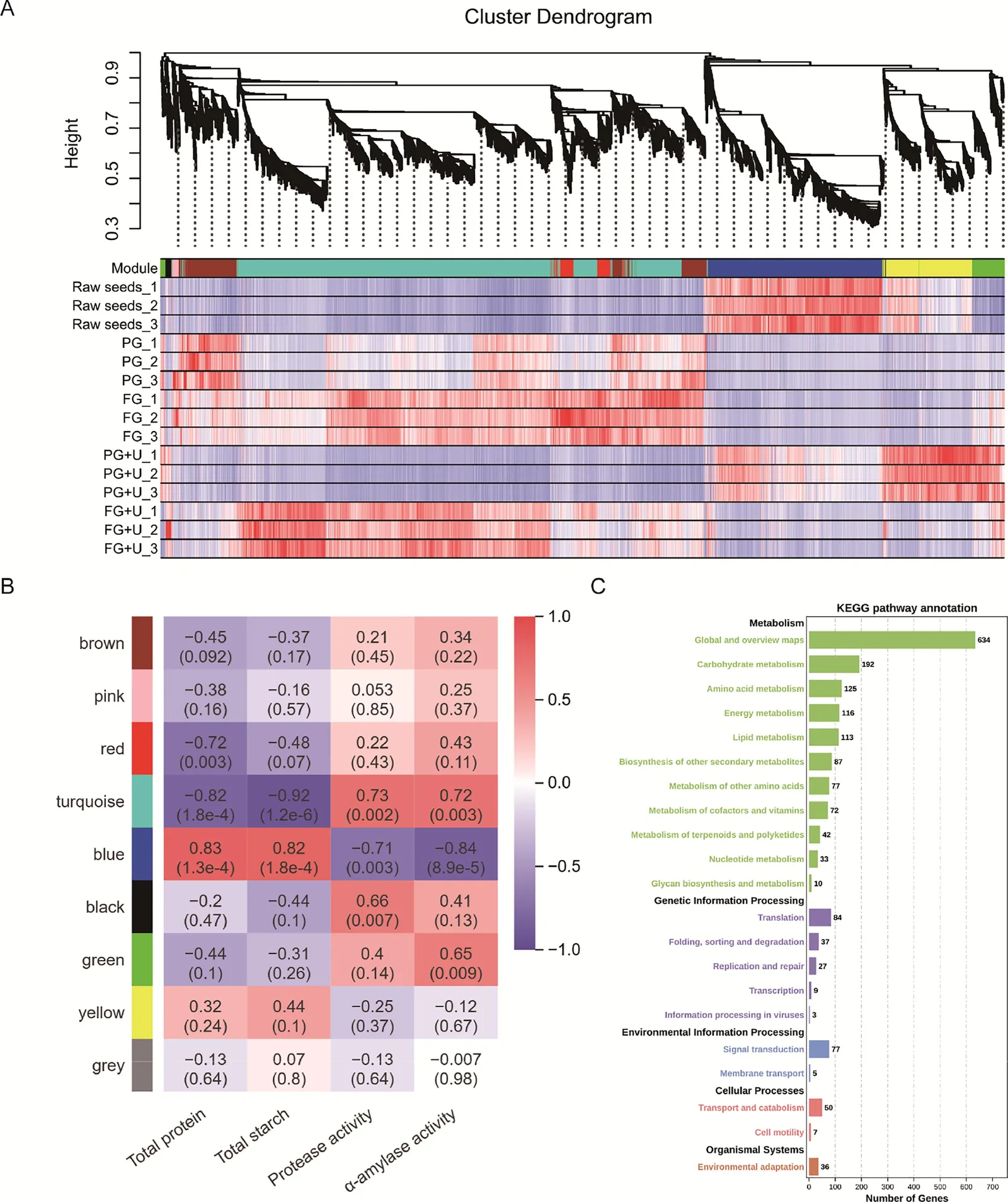
WGCNA and KEGG pathway analyses. (A) Hierarchical clustering dendrogram of genes based on WGCNA across raw seeds, PG, FG, PG + U, and FG + U groups. (B) Heatmap of module–trait relationships for total protein content, total starch content, protease activity, and α-amylase activity. (C) KEGG pathway enrichment analysis of the turquoise module.

To investigate gene co-expression patterns associated with ultrasound-assisted sorghum germination, WGCNA was performed using 7,791 DEGs. As shown in Fig. 4A, these DEGs were grouped into nine distinct co-expression modules, each representing a cluster of highly correlated genes. Genes within the same module exhibited similar expression patterns, reflecting coordinated gene expression. Correlation analysis between module eigengenes and metabolic traits, including total protein and total starch contents, revealed clear module–trait relationships (Fig. 4B). The blue module exhibited relatively strong positive correlations with total protein and total starch contents (r = 0.83 and 0.82, respectively; both *p* < 0.001). These positive correlations indicate that genes within the blue module are associated with storage reserve accumulation during sorghum germination. The blue module showed significant negative correlations with protease activity (r =  − 0.71, *p* = 0.003) and α-amylase activity (r =  − 0.84, *p* < 0.001), suggesting a weaker association of this module with reserve mobilization. The turquoise module (3,744 genes) showed the strongest negative correlations with both total protein and total starch contents (r =  − 0.82 and − 0.92, respectively; both *p* < 0.001), suggesting that genes within the turquoise module were associated with starch and protein hydrolysis during sorghum germination. This module displayed significant positive correlations with protease activity (r = 0.73, *p* = 0.002) and α-amylase activity (r = 0.72, *p* = 0.003), further supporting its involvement in enzymatic degradation of storage reserves during germination. Bootstrap analysis and leave-one-out sensitivity analysis confirmed the robustness and stability of the correlations between the turquoise module eigengene and physiological traits (Table S3 and S4).

To further characterize the turquoise module, functional annotation based on KEGG pathways was carried out using the genes assigned to this module (Fig. 4C). The annotated genes were predominantly enriched in metabolic pathways, indicating extensive metabolic activity. Within the Metabolism category, global and overview maps accounted for the largest proportion of annotated genes, followed by carbohydrate metabolism, amino acid metabolism, energy metabolism, lipid metabolism, secondary metabolite biosynthesis, and cofactor metabolism, indicating coordinated metabolic activity across multiple biological processes during germination. Genes associated with Genetic Information Processing were primarily involved in translation and protein turnover, with fewer genes associated with replication and transcription. Among the enriched pathways, carbohydrate metabolism and amino acid metabolism were particularly prominent. These findings provided the basis for the subsequent identification of candidate genes and transcription factors associated with the enriched metabolic processes.

### 3.6 Integrated transcription–metabolite association network analysis

**Fig. 5:**
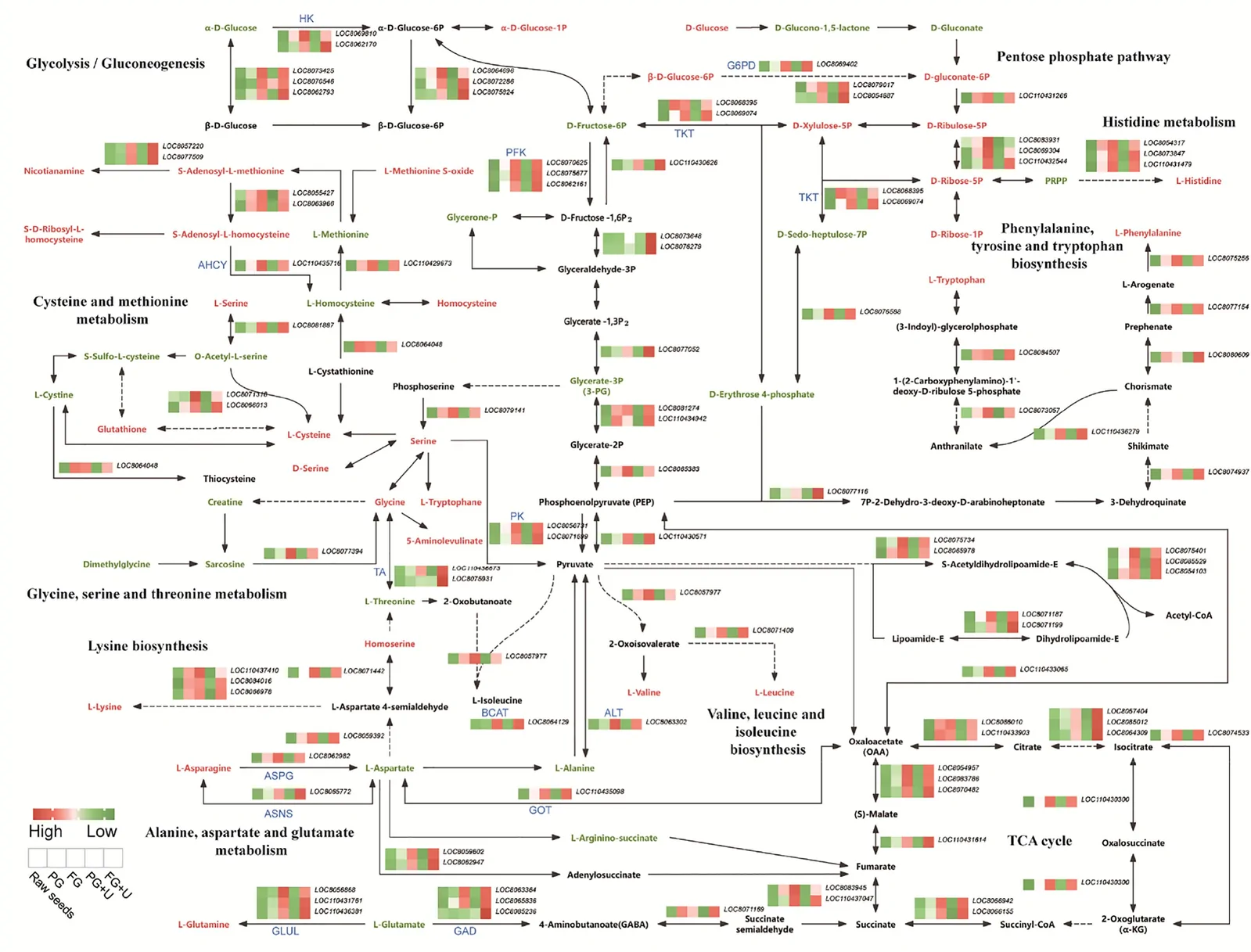
Integrated transcription-metabolite association network of central amino acid and carbohydrate metabolism. Red and green indicate up- and down-regulated metabolites, respectively. Solid and dashed lines indicate direct and multi-step reaction routes, respectively. Gene expression levels are shown as a color gradient from low (green) to high (red). Hexokinase (HK), transketolase (TKT), phosphofructokinase (PFK), alanine aminotransferase (ALT), aspartate aminotransferase (GOT), glucose-6-phosphate dehydrogenase (G6PD), glutamine synthetase (GLUL), branched-chain amino acid aminotransferase (BCAT), glutamate decarboxylase (GAD), asparaginase (ASPG), pyruvate kinase (PK), threonine aldolase (TA), s-adenosylhomocysteine hydrolase (AHCY), and asparagine synthetase (ASNS).

To elucidate the associations between gene expression and metabolite accumulation, an integrated transcription–metabolite association network was constructed by combining transcriptomic and metabolomic datasets (Fig. 5). The integrated multi-omics analysis revealed coordinated changes in differentially expressed genes and differential metabolites, with major alterations associated with carbohydrate and amino acid metabolism.

In glycolysis and the pentose phosphate pathway, ultrasonic treatment was associated with changes in both key metabolic genes and associated metabolites. The gene *LOC8062170*, encoding hexokinase (HK), which catalyzes the phosphorylation of glucose to glucose-6-phosphate, was significantly upregulated following ultrasonic treatment. The increased expression of HK, together with concurrent changes in carbohydrate-related metabolites, suggested coordinated changes in glucose phosphorylation-related processes following ultrasonic treatment. Increased levels of α-D-glucose-1-phosphate were also observed, further supporting an association between ultrasonic treatment and changes in starch- and sucrose-related metabolism. Similarly, phosphofructokinase (PFK), which catalyzes the conversion of D-fructose-6-phosphate to D-fructose-1,6-bisphosphate, was affected at the transcriptional level, with multiple PFK-encoding genes (*LOC8070625*, *LOC8075677*, and *LOC806216*1) significantly upregulated under ultrasonic treatment. This transcriptional change was accompanied by decreased D-fructose-6-phosphate levels, suggesting changes in glycolytic metabolite distribution. Furthermore, genes encoding glucose-6-phosphate dehydrogenase (G6PD; *LOC8069402*) and transketolase (TKT; *LOC8069074*) were upregulated following ultrasonic treatment. The increased abundance of metabolites associated with the pentose phosphate pathway, including D-xylulose-5-phosphate, D-gluconate-6-phosphate, D-ribulose-5-phosphate, D-ribose-5-phosphate, and D-ribose-1-phosphate, was consistent with alterations in pentose phosphate pathway-related metabolism following ultrasonic treatment.

Ultrasonic treatment was also associated with changes in amino acid metabolism, with 21 metabolites associated with amino acid pathways showing upregulation, including 16 amino acids with increased accumulation. The coordinated alterations in amino acid-related transcripts and metabolites suggested broad changes in amino acid metabolism following ultrasonic treatment. In alanine, aspartate, and glutamate metabolism, the genes *LOC110431761* and *LOC110436381* encoding glutamine synthetase (GLUL) were significantly upregulated, and this expression pattern was associated with increased glutamine accumulation. Similarly, asparagine synthetase (ASNS; *LOC8065772*) was upregulated, which was correlated with decreased L-aspartate and increased L-asparagine levels, suggesting a potential association between ASNS expression and asparagine metabolism. In glycine, serine, and threonine metabolism, the genes *LOC8070531* and *LOC110436673* encoding threonine aldolase (TA) were significantly upregulated under ultrasonic treatment. This transcriptional change coincided with increased glycine and decreased threonine levels, indicating that TA-related expression changes may participate in altered glycine metabolism. These results suggest that TA-associated metabolic responses may be associated with broader changes in amino acid metabolism, including alterations in serine, tryptophan, and cysteine metabolism.

### 3.7 Transcription factor–target gene network analysis

To construct the TF (transcription factor)–target gene co-expression network, genes from the turquoise module identified by WGCNA were analyzed, resulting in a network comprising 32 TFs, 150 target genes, and 841 interaction edges (Fig. S6). These 32 TFs belonged to 15 TF families, including bHLH, MYB, WRKY, bZIP, and NAC. Several TF families, particularly bHLH and MYB, were highly represented in the network and were significantly associated with total starch and total protein contents, suggesting their potential involvement in carbohydrate and amino acid metabolic processes during germination and ultrasonic treatment. Within the network, the bHLH transcription factors bHLH137 (*LOC8060932*) and R-S (*LOC8056590*) exhibited the highest connectivity, interacting with 67 and 63 target genes, respectively. These highly connected TFs were identified as candidate hub transcription factors based on network topology. To further validate the RNA-seq results, four highly connected TFs were selected from the co-expression network, and three TFs showed significant upregulation in ultrasound-treated germinated sorghum. The expression patterns of these candidate hub TFs showed high consistency with the RNA-seq results (Fig. S7), supporting their potential involvement in ultrasound-responsive regulatory networks.

## 4 Discussion

Sorghum is a widely cultivated cereal crop of considerable nutritional and economic importance, serving as a staple food for human consumption and an important feed resource for livestock production in many regions worldwide [28]. As an edible seed, sorghum grains are rich in carbohydrates and proteins, which are essential for human and animal nutrition [29]. Germination improves the nutritional quality of cereal grains by converting macromolecules into low-molecular-weight compounds [30]. In this study, germination significantly reduced total protein and starch contents compared with raw seeds, while ultrasonic treatment increased the activities of protease and α-amylase during germination. These findings suggest that ultrasound may facilitate reserve mobilization during germination, which could contribute to improved germination performance and early seedling development. Ultrasound-assisted germination has been reported to accelerate seed metabolism and promote nutrient mobilization and is therefore widely applied to improve germination efficiency and bioactive compound accumulation in edible seeds [16]. Previous investigations have generally examined transcriptional and metabolic responses separately rather than in an integrated framework, limiting a system-level understanding of the underlying regulatory mechanisms. Therefore, a WGCNA-guided multi-omics approach was employed to systematically characterize transcriptional and metabolic regulation of amino acid and carbohydrate metabolism during ultrasound-assisted germination in sorghum.

Metabolomic analysis demonstrated that ultrasound-assisted germination induced pronounced alterations in the metabolic profile of sorghum grains at the fully germinated stage, particularly affecting amino acid and carbohydrate metabolism. Changes in amino acid metabolism are of particular nutritional relevance. The increased accumulation of free amino acids suggests enhanced mobilization of protein reserves under ultrasound treatment, which may improve nutritional availability. Gong et al. [31] reported that ultrasound significantly improved germination performance and protease activity in maize seeds, indicating enhanced protein degradation and nitrogen mobilization. Similarly, ultrasound treatment in soybean has been shown to accelerate germination, enhance enzyme activities, and stimulate metabolic pathways associated with protein turnover and phenylalanine metabolism [16]. In this study, fully germinated sorghum grains subjected to ultrasound (FG + U) showed higher levels of amino acids and derivatives than FG, including essential amino acids such as L-phenylalanine, L-lysine, L-valine, L-isoleucine, L-leucine, and L-tryptophan. Compared with previous studies, this metabolomic analysis further characterized changes in the accumulation of multiple essential amino acids. Essential amino acids (EAAs), which cannot be synthesized in humans, are required for protein synthesis, tissue growth, and metabolic function and are widely used as indicators of protein nutritional quality [32]. The increased accumulation of these essential amino acids suggests the potential for improved nutritional value, although further studies evaluating digestibility, bioavailability, and other nutritional attributes are required to confirm this possibility. Among these metabolites, L-phenylalanine, a precursor of phenylpropanoid biosynthesis, also showed increased accumulation, suggesting changes in secondary metabolic processes. Phenylpropanoid glycosides (PPGs) are well-documented for their antioxidant, anti-inflammatory, antibacterial, antitumor, and antiplatelet activities [33]. Similarly, L-glutamine, a key metabolite in nitrogen metabolism, was significantly increased under ultrasound treatment, indicating enhanced nitrogen remobilization and amino acid biosynthesis activity. L-glutamine, which represents the most abundant free amino acid circulating in the human body, plays essential roles in intestinal integrity, immune function, nitrogen transport, and cellular biosynthesis, contributing to metabolic homeostasis [34]. Ultrasound treatment also significantly influenced carbohydrate metabolism in fully germinated sorghum grains. Stachyose, a raffinose-family oligosaccharide (RFO) involved in carbohydrate storage and transport [35], showed reduced abundance, whereas D-glucose, a central monosaccharide entering glycolysis and the pentose phosphate pathway, was increased [36]. Compared with these previous studies, this metabolomic analysis further identified specific changes in carbohydrate metabolites, including decreased stachyose and increased D-glucose, providing further insight into carbohydrate metabolic alterations during ultrasound-assisted germination. These findings suggest that ultrasound may facilitate the conversion of storage carbohydrates into readily metabolizable sugars, enhancing carbon flux toward energy-producing pathways. The increased D-glucose content provides readily fermentable substrates for microbial metabolism, which may enhance fermentation efficiency and contribute to the formation of desirable flavor-active metabolites in sorghum-based fermented foods and beverages [37]. Enhanced carbohydrate metabolism reflects more efficient mobilization of starch reserves, providing energy and carbon skeletons to support amino acid biosynthesis and other metabolic activities during germination [38]. These findings highlight the potential of ultrasound-assisted germination as a promising strategy for enhancing grain germination and accelerating amino acid and carbohydrate metabolism.

Transcriptomic analysis provided molecular insights into the transcriptional responses associated with these metabolic changes. Seed germination is a highly coordinated process involving extensive transcriptional reprogramming, particularly in genes associated with mobilization of storage reserves such as starch and proteins [39]. In this study, amino acid metabolism pathways, particularly alanine, aspartate, and glutamate metabolism, were responsive to ultrasound treatment. Upregulation of *LOC8065772*, *LOC110431761*, and *LOC110436381* was associated with increased accumulation of L-asparagine and L-glutamine. This is consistent with previous findings in quinoa, where ultrasound stress significantly regulated amino acid-related pathways, especially those involving alanine-family amino acids, and exhibited pronounced responsiveness to ultrasonic treatment, as well as nitrogen metabolism, affecting amino acid biosynthesis and interconversion [40]. Similarly, glycine, serine, and threonine metabolism was affected by ultrasound treatment. Upregulation of *LOC8070531* and *LOC110436673* was accompanied by increased glycine accumulation. These results suggest that ultrasound is associated with coordinated changes in amino acid metabolic pathways, which may contribute to amino acid biosynthesis and interconversion. Pathways central to carbohydrate catabolism and anabolism, particularly glycolysis/gluconeogenesis and the pentose phosphate pathway, also showed significant responses to ultrasound treatment, indicating enhanced carbon flux toward energy production and metabolic intermediates required for germination. Glycolysis is considered a pivotal metabolic process during seed germination, as it not only supplies ATP to meet the increasing energy demand of germinating seeds but also provides carbon skeletons and intermediate metabolites necessary for cellular biosynthesis and seedling development [38]. Upregulation of *LOC8062170* was associated with increased accumulation of D-glucose-1-phosphate. Upregulation of *LOC8069402* and *LOC8069074* was associated with increased levels of D-gluconate-6-phosphate and D-xylulose-5-phosphate, suggesting enhanced pentose phosphate pathway activity. This observation is consistent with increased G6PD expression, which may be associated with enhanced NADPH production and the availability of metabolic precursors [41]. Similar effects of ultrasound on carbohydrate metabolism and soluble sugar accumulation have been documented in various other plant systems [42].

Transcription factor analysis suggested that members of the bHLH family may be involved in metabolic reprogramming. Among identified transcription factors, bHLH137 (*LOC8060932*) and R-S (*LOC8056590*) showed the highest connectivity in the TF–target gene co-expression network, suggesting that they represent candidate hub transcription factors associated with amino acid and carbohydrate metabolism rather than confirmed regulators. bHLH transcription factors have been implicated in diverse plant metabolic processes. For example, *bHLH137* is involved in MBW complex-mediated regulation of anthocyanin biosynthesis in eggplant [43], indicating broad regulatory potential in secondary metabolism. R/B-type bHLH proteins are also key components of MBW complexes regulating plant metabolic pathways [44]. These previous studies provide evidence that bHLH members can participate in metabolic regulation; however, whether bHLH137 and R-S directly regulate ultrasound-responsive metabolic pathways in sorghum requires further functional validation. Consistent with previous observations, including those from our earlier study, ultrasound-enhanced germination in brown rice is associated with altered expression of bHLH transcription factors linked to carbohydrate and amino acid metabolism [26]. Several bHLH subfamilies, particularly MYC and PIF proteins, are known regulators of primary metabolism [45]. MYC3 and MYC4 regulate jasmonate-induced tryptophan metabolism in *Arabidopsis thaliana* [46], while NtMYC2a influences starch accumulation and sugar conversion during pollen maturation in tobacco [47]. PIF4 has also been reported to regulate carbohydrate metabolism by modulating starch utilization in *Arabidopsis* [48]. These studies support the possibility that bHLH transcription factors contribute to metabolic adjustment during plant development and stress responses. In this study, ultrasound treatment was associated with increased expression of the putative hub transcription factors bHLH137 and R-S, together with changes in metabolites such as L-asparagine, L-glutamine, D-glucose-1-phosphate, and D-gluconate-6-phosphate. These transcriptional and metabolic changes may contribute to altered amino acid accumulation and carbohydrate utilization during germination. However, the implications of these changes for the nutritional quality of germinated sorghum require further validation.

The physiological mechanisms underlying ultrasound-enhanced germination are likely multifaceted. Although this study primarily focused on reserve mobilization and transcriptional-metabolic regulation, changes in endogenous phytohormone homeostasis and antioxidant enzyme activities were not directly investigated. Previous studies have suggested that ultrasound may promote seed germination by modulating these physiological processes [49]. Further investigation of the relationships among phytohormone signaling, antioxidant responses, reserve mobilization, and bioactive compound accumulation would provide a more comprehensive understanding of the physiological and molecular responses associated with ultrasound-assisted germination and their potential implications for grain quality. Moreover, functional experiments, including gene overexpression or silencing, ChIP-qPCR, and dual-luciferase reporter assays, are required to validate the candidate regulatory genes and transcriptional networks identified in this study.

## 5 Conclusion

Ultrasound-assisted germination was associated with improved sorghum sprout growth and enhanced hydrolysis of storage proteins and starch, indicating increased reserve mobilization during germination. Ultrasound treatment was also associated with increased accumulation of nutritionally relevant amino acids and D-glucose, accompanied by enrichment of genes involved in amino acid and carbohydrate metabolism. Integrated transcriptomic and metabolomic analyses further indicated that these metabolic alterations were associated with coordinated changes in gene expression, providing systems-level insight into the transcriptional and metabolic responses during ultrasound-assisted germination. WGCNA combined with transcription factor–target gene network analysis identified three highly connected transcription factors, including bHLH137 (*LOC8060932*), R-S (*LOC8056590*), and EIL1 (*LOC110434014*), which were supported by RT-qPCR validation as candidate hub transcription factors associated with the observed transcriptional responses. These results provide a base for further studies aimed at functionally validating the proposed regulatory relationships, optimizing ultrasound treatment conditions, and evaluating the potential application of ultrasound-assisted germination for the production of nutritionally improved sorghum-based foods.

## CRediT authorship contribution statement

**Qiao Huang:** Writing – original draft, Methodology, Formal analysis, Data curation, Conceptualization. **Yang Liu:** Formal analysis, Data curation. **Yiqiao Wang:** Visualization, Methodology. **Yiting Zhao:** Visualization. **Jiarong Liu:** Software. **Tao Xu:** Investigation. **Yutang He:** Investigation. **Guangchen Zhang:** Writing – review & editing, Supervision, Resources, Funding acquisition. **He Liu:** Supervision, Resources, Funding acquisition.

## Declaration of competing interest

The authors declare that they have no known competing financial interests or personal relationships that could have appeared to influence the work reported in this paper.

## Appendix A Supplementary data

Supplementary Data 1 Supplementary figures and tables supporting the experimental, metabolomic, transcriptomic, and statistical analyses.
