# Supplementary material for: Ultrasound-Assisted Enhancement of Germinated Sorghum: Multi-Omics Insights into Amino Acid and Carbohydrate Metabolic Regulation
Source: Ultrason Sonochem. 2026 Aug 19;133:108013. doi: 10.1016/j.ultsonch.2026.108013 (PMC13524591; doi:10.1016/j.ultsonch.2026.108013)
Supplement: Supplementary file 1 [file mmc1.docx]

**Supplementary material**

Fig. S1. Effect of ultrasound treatment on germination length of fully germinated sorghum samples (FG and FG+U). Differences between FG and FG+U within each cultivar were analysed using an unpaired two-tailed Student's *t*-test. Data are presented as mean ± SEM (n = 10). * *p* < 0.05, ** *p* < 0.01, *** *p* < 0.001.

Fig. S2. Changes in (A) protease activity and (B) α-amylase activity during germination among different treatment groups (raw seeds, PG, PG+U, FG, and FG+U). Data are presented as mean ± SD (n = 3). Different letters indicate significant differences at *p* < 0.05.

Fig. S3. Permutation test validation of the OPLS-DA model for FG+U versus FG sorghum samples. The OPLS-DA model was validated using 200 permutation tests. The original model showed R²Y = 1.000 and Q² = 0.987. The Q² intercept was −0.436 (R²Y intercept = 0.978). A negative Q² intercept indicates that the model was not overfitted.

Fig. S4. Hierarchical clustering heatmaps of 182 amino acids (A) and 99 carbohydrates (B) across FG and FG+U groups.

Fig. S5. Validation of RNA-seq results by RT-qPCR. Data are presented as mean ± SD (n = 3).

Fig. S6. Transcription factor (TF) family distribution and co-expression network analysis. (A) Top 10 transcription factor families identified in the turquoise module identified by WGCNA. (B) TF-target gene co-expression network derived from the turquoise module identified by WGCNA. Nodes represent TFs and target genes, with node size proportional to the number of connections (degree). Transcription factors and target genes are connected by undirected edges. Colored nodes denote different TF families and their associated target genes.

Fig. S7. qRT-PCR validation of ultrasound-responsive expression patterns of four candidate transcription factors across six sorghum varieties. Data are presented as mean ± SD (n = 3). ns, not significant (*p* ≥ 0.05); * *p* < 0.05, ** *p* < 0.01, *** *p* < 0.001.

Table S1. Primer sequence used for RT-qPCR analysis.

| Gene ID | Forward primer（5'→3'） | Reverse primer（5'→3'） |
| --- | --- | --- |
| Randomly selected gene validation | | |
| *Actin* | ATGTTTCTTCATGTAGAACATCGAT | TTCCAGCAGATGTGGATCTCCAAG |
| *LOC8070625* | TGGTGTCCACATGCAGCAGAAG | CCTGACGTGATGCCACTGAACC |
| *LOC8073875* | ACGGCCTCAGAGCGAGCAAT | TGTTCAGGTCCAGCAGCAGCA |
| *LOC8055635* | GTCGGCATCGCTGACTTCAACT | ACATCGCCACGGTCGGTATGA |
| *LOC110431479* | GCATTGGTTCTGTCGGGCTGTA | ACGGCTAGGTGGTGTGGCAA |
| *LOC110435716* | GACGCAACGCTGCTCATCCA | ACGCCGACAAGCCTCTCCTT |
| Transcription factor validation | | |
| *Actin* | ATGTTTCTTCATGTAGAACATCGAT | TTCCAGCAGATGTGGATCTCCAAG |
| *LOC8060932* | AGCACCACTGAAGATGAGGC | TCTCTCCTCACCCTCTCTGC |
| *LOC8056590* | GCTATGCCCTCTTCTGGTCC | TCACCTCGCCGTTGTAGAAC |
| *LOC8072434* | ACCTCCTGCAGTTCTCCGA | CCACTCCAGCTCCTTGAACC |
| *LOC110434014* | TGGAAGGAGTTGCCGTGAAG | GGAAGGTGGGGAAGGTCATG |

Table S2. Summary of RNA sequencing and alignment data for each sample.

| Sample | Raw Reads | Raw Bases | Clean Reads | Clean Bases | Error Rate(%) | Q20(%) | Q30(%) | GC Content(%) |
| --- | --- | --- | --- | --- | --- | --- | --- | --- |
| Raw seeds-1 | 46117784 | 6.92G | 43403316 | 6.51G | 0.03 | 96.87 | 92.18 | 56.06 |
| Raw seeds-2 | 42973892 | 6.45G | 41236772 | 6.19G | 0.03 | 96.76 | 91.82 | 56.19 |
| Raw seeds-3 | 46354242 | 6.95G | 44249668 | 6.64G | 0.03 | 96.69 | 91.66 | 55.37 |
| PG-1 | 49967150 | 7.5G | 48326396 | 7.25G | 0.03 | 97.09 | 92.39 | 52.17 |
| PG-2 | 44302094 | 6.65G | 42952652 | 6.44G | 0.03 | 97.25 | 92.73 | 52.49 |
| PG-3 | 42733840 | 6.41G | 40208610 | 6.03G | 0.03 | 97.37 | 92.99 | 51.95 |
| PG+U-1 | 45896114 | 6.88G | 44631524 | 6.69G | 0.03 | 96.78 | 91.85 | 55.43 |
| PG+U-2 | 45797618 | 6.87G | 42703674 | 6.41G | 0.03 | 96.52 | 91.31 | 54.03 |
| PG+U-3 | 45444642 | 6.82G | 43261168 | 6.49G | 0.03 | 96.72 | 91.7 | 53.79 |
| FG-1 | 43480962 | 6.52G | 42196470 | 6.33G | 0.03 | 96.93 | 92.13 | 55.03 |
| FG-2 | 44993684 | 6.75G | 43644810 | 6.55G | 0.03 | 96.97 | 92.23 | 55.32 |
| FG-3 | 43462100 | 6.52G | 41543324 | 6.23G | 0.03 | 97.07 | 92.43 | 54.51 |
| FG+U-1 | 45799110 | 6.87G | 43637410 | 6.55G | 0.03 | 96.98 | 92.19 | 53.37 |
| FG+U-2 | 45578198 | 6.84G | 43395982 | 6.51G | 0.03 | 96.94 | 92.11 | 52.8 |
| FG+U-3 | 51237726 | 7.69G | 50061032 | 7.51G | 0.03 | 97.29 | 92.88 | 52.8 |

Table S3. Correlation analysis between the MEturquoise module and total protein, total starch, protease activity, and α-amylase activity

| Variable | Pearson r | *p*-value | Bootstrap 95% CI (BCa) | Spearman ρ |
| --- | --- | --- | --- | --- |
| ME-Total protein | -0.823 | <0.001 | (-0.885, -0.766) | -0.786 |
| ME-Total starch | -0.917 | <0.001 | (-0.925, -0.910) | -0.963 |
| ME-Protease activity | 0.73 | 0.002 | (0.589, 0.878) | 0.85 |
| ME-α-amylase activity | 0.723 | 0.002 | (0.656, 0.786) | 0.875 |

Bootstrap confidence intervals were calculated for Pearson correlation coefficients using 2000 resamples with BCa correction.

Table S4. Leave-one-out sensitivity analysis of the correlation stability between the MEturquoise module and physiological traits.

| Removed sample | r(MEturquoise–Protein) | r(MEturquoise–Starch) | r(MEturquoise–Protease activity) | r(MEturquoise–α-amylase activity) |
| --- | --- | --- | --- | --- |
| Raw seeds-1 | -0.805015699 | -0.91156008 | 0.700480551 | 0.689736428 |
| Raw seeds-2 | -0.80350073 | -0.90958591 | 0.705967215 | 0.687894524 |
| Raw seeds-3 | -0.80126032 | -0.907739686 | 0.694757015 | 0.690813623 |
| PG-1 | -0.83309474 | -0.922368633 | 0.734904425 | 0.723751397 |
| PG-2 | -0.82704002 | -0.919553471 | 0.740145615 | 0.729280797 |
| PG-3 | -0.823396382 | -0.919898957 | 0.730447612 | 0.723144248 |
| PG+U-1 | -0.863683073 | -0.930219821 | 0.729513833 | 0.80998211 |
| PG+U-2 | -0.877775284 | -0.930504078 | 0.805085304 | 0.767755458 |
| PG+U-3 | -0.820690574 | -0.93235454 | 0.75638058 | 0.783548921 |
| FG-1 | -0.810736137 | -0.918016758 | 0.754868655 | 0.713278427 |
| FG-2 | -0.807149998 | -0.916402122 | 0.765231188 | 0.707882567 |
| FG-3 | -0.818023469 | -0.914967228 | 0.717571431 | 0.718253296 |
| FG+U-1 | -0.820591636 | -0.905442123 | 0.70206452 | 0.685410299 |
| FG+U-2 | -0.821043282 | -0.908653801 | 0.712399047 | 0.701518158 |
| FG+U-3 | -0.815556114 | -0.910135849 | 0.707253903 | 0.70579797 |
